# Time from sudden sensory neural hearing loss to treatment as a prognostic factor

**DOI:** 10.3389/fneur.2023.1158955

**Published:** 2023-04-14

**Authors:** Itay Chen, Shalom Eligal, Ori Menahem, Riki Salem, Jean-Yves Sichel, Ronen Perez, Chanan Shaul

**Affiliations:** Department of Otolaryngology and Head and Neck Surgery, Shaare-Zedek Medical Center, Faculty of Medicine, Hebrew University of Jerusalem, Jerusalem, Israel

**Keywords:** hearing loss, corticosteorids, audiogram, intratymapnic injection, discrimination

## Abstract

**Introduction:**

The widely accepted treatment for sudden sensorineural hearing loss (SSNHL) is corticosteroid treatment (oral or intratympanic). The main goal of this work is to define the significance of the time between symptom onset and treatment initiation, as well as other prognostic factors, for hearing improvement.

**Methods:**

This retrospective study included 666 patients treated for SSNHL. Demographic data, audiometry, treatment method, time since symptom onset, and associated symptoms were recorded for each patient. The patients were divided into five groups according to the treatment initiation time—half a week, one week, 2 weeks, 3 weeks, or 4 weeks and over—after symptom onset. The degree of improvement was assessed by comparing the audiometry at the beginning and the end of the treatment.

**Results:**

The average period of hearing loss from symptom onset to treatment initiation was 10.8 days. Significant differences were found between the groups of half a week, one week, and 2 weeks and the groups of 3 weeks and 4 weeks and over (each separately, *p* < 0.001). No difference was found between the half-week, one-week, and two-week groups, nor was there a difference between the three-week and four-week-and-over groups. A correlation was found between the treatment initiation time in days and the degree of improvement in hearing for both speech recognition threshold (SRT) and discrimination, *R* = 0.26 *p* < 0.001 and *R* = 0.17 *p* < 0.001, respectively. No correlation was found for gender, age of the patients, comorbidities, or associated symptoms.

**Conclusion:**

The threshold for treatment initiation time is up to 2 weeks, after which the amplitude of hearing improvement decreases significantly. The other prognostic factors measured were not found to be statistically significant predictors.

## 1. Introduction

Sudden sensory neural hearing loss (SSNHL) is defined as sensory neural hearing loss that appears within 72 h and is manifested by a decrease of at least 30 decibels (dB) in three consecutive frequencies in audiometry (1). The annual incidence of SSNHL is 5–27 people per 100,000 (2), with a 32–65% chance of spontaneous recovery without treatment (3–6). Several prognostic factors have been identified, including the patient’s age, the degree of hearing loss (HL), and additional symptoms (e.g., tinnitus, vertigo) (6).

Oral treatment with corticosteroids (CTS) with varying periods and dosages is the recommended treatment nowadays. The latest guidelines of the American Academy of Otolaryngology (AAO) define treatment with oral CTS as an optional treatment with a moderate level of evidence (7). According to these guidelines, the recommended period of time for initiation of treatment with oral CTS is up to 14 days from symptom onset. Academia and the literature base this recommendation on laboratory evidence of an inflammatory cell death cascade in SSNHL; the CTS aims to stop this cascade, and the window of time is set to 14 days (7, 8).

It is natural to assume that these laboratory findings will have clinical consequences. However, there is no consensus in the literature as to whether there is a strong correlation between the time from symptom onset to initiation of treatment and hearing improvement. Fetterman et al. did not find a clear relationship between these two factors and therefore did not include treatment initiation time as a prognostic factor (9). In contrast, Cvorovic et al. found that initiating CTS treatment within 7 days of symptom onset has a better prognosis than initiating treatment later (10). Those studies looked for a correlation between the time from onset of HL to treatment initiation but did not directly compare patients who began treatment before and after 14 days. Furthermore, the AAO recommended that clinicians should offer intratympanic steroid therapy when patients have incomplete recovery from SSNHL, even two to 6 weeks after symptom onset (7).

So far, no comprehensive data have been published to evaluate the AAO guidelines and recommendations, particularly the precise onset of oral treatment (up to 14 days). The main objective of this paper is to determine the relationship between the time from symptom onset to initiation of CTS treatment and improvement in hearing among patients suffering from SSNHL. The secondary objectives are to investigate additional prognostic factors (e.g., smoking, ischemic heart disease) (IHD), diabetes mellitus (DM), hypertension (HTN), age, gender, the severity of HL, and accompanying symptoms (tinnitus, vertigo) and their degree of correlation with improvement under CTS treatment.

## 2. Materials and methods

A retrospective cohort study was conducted on patients admitted to the Department of Otolaryngology and Head–Neck Surgery at Shaare Zedek Medical Center, diagnosed with SSNHL, and hospitalized during 2012–2021. The institutional review board approved the study protocol with a waiver of informed consent.

The patients’ general information was collected, including demographics, medical background (IHD, DM, HTN), medications, accompanying symptoms, and the time from symptom onset to initiation of treatment. At least two hearing tests were performed for each patient (before and at the end of the treatment). These tests measured pure tone audiometry (PTA), Speech Recognition Threshold (SRT), and discrimination.

The treatment protocol followed the AAO Head and Neck Surgery Guidelines; that is, with no contraindication to oral corticosteroids (OCS), all patients were treated with Prednisone 30 mg twice daily for 1 week, and if no sufficient improvement was seen (if there was still ≥10 dB sensorineural hearing loss in at least two frequencies) a salvage treatment with once-daily intratympanic dexamethasone injection was initiated for another week while tapering the OCS over 5 days (7).

Inclusion criteria were presentation with SSNHL; exclusion criteria were partial treatment, a different diagnosis from SSNHL (conductive HL, acoustic trauma, vestibular schwannoma, Meniere’s disease), congenital HL, and failure to follow-up.

The final cohort of patients enrolled was divided into five groups according to the period of time from symptom onset to treatment initiation as follows: 1st group: up to half a week (1–3 days) from onset of HL; 2nd group: 4–7 days, 3rd group: 8–14 days, 4th group: 15–21 days, 5th group: 22 days and over.

### 2.1. Audiometry tests

Certified audiologists in our medical center performed audiometry. The tests were performed in soundproof booths using a Grason-Stadler (GSI-61/AudioStar Pro) audiometer (Minnesota, USA) with standard audiometric parameters. The audiometers were calibrated annually. Pure tone average (PTA) was calculated using 500, 1,000, and 2,000 Hz. Maximum speech discrimination score % (SD) and speech recognition thresholds (SRT) were included for analysis; SRT is the minimum hearing level at which an individual can recognize 50% of spondaic words. The maximum speech discrimination score was obtained at a level of 35 dB above the SRT, or at a softer level if the standard level exceeded the users’ comfort level or maximum output of the audiometer. A list of 50 monosyllabic Hebrew words was presented mostly in the live voice condition, and the maximum score was determined as the percentage of words repeated correctly.

### 2.2. Data processing

The effectiveness of the treatment was measured by calculating the improvement in specific frequencies, SRT, and discrimination for each individual. The amplitude of HL was determined both absolutely by considering only the affected ear and relatively by comparing the HL of the affected ear to the healthy ear (given that the healthy ear was not damaged), as follows:

Absolute: comparison between affected ear at the end of treatment (AFFend) and affected ear before treatment (AFFbef).

The equation used for absolute SRT measurements:

AFFend SRT - AFFbef SRT

Relative: comparison between affected ear improvement and severity of HL. This was calculated by dividing the difference between AFFend and AFFbef by the relative HL (i.e., healthy ear minus AFFbef).

The equation used for relative SRT measurements:


$$\frac{\mathrm{AFFend}\mathrm{SRT}-\mathrm{AFFbef}\mathrm{SRT}}{\mathrm{Healthy}\mathrm{SRT}-\mathrm{AFFbef}\mathrm{SRT}}$$

### 2.3. Statistical analysis

All data were collected in spreadsheets using Microsoft Excel. The statistical analysis was performed using SPSS software (IBM^®^ SPSS^®^ Statistics, version 26, Chicago, IL, United States). The statistical comparison between the different groups (divided according to time from symptom onset to initiation of treatment) was made using the non-parametric Kruskal-Wallis test; in the case where a statistically significant difference was found among all groups, we used the Mann–Whitney test to compare any two different groups with Bonferroni correction for multiple tests.

All statistical tests were two-tailed. Statistical significance was defined as *p* ≤ 0.05. The degree of correlation between the time from symptom onset to treatment initiation and other possible prognostic factors with the success of the treatment was tested using the Spearman correlation test, where statistical significance was defined as *p* ≤ 0.05.

## 3. Results

During the period 2012–2021, 765 patients diagnosed with SSNHL were hospitalized at Shaare Zedek Medical Center. After reviewing the files, 99 patients were excluded from the initial cohort in accordance with the exclusion criteria.

The study’s final cohort included 666 patients: 336 men and 330 women. Right ear SSNHL was present in 329 patients, while 324 patients presented with left ear SSNHL, and 13 patients presented with bilateral SSNHL. The demographic data, medical background, and accompanying SSNHL symptoms are presented in Table 1. No significant statistical difference was found among the five time groups for any of the above parameters.

**Table 1:** Demographic information, including gender, comorbidities, and associated symptoms, as a function of time from symptom onset.

Weeks	0.5	1	2	3	4+	*p*
Number	202	177	159	61	67	
M/F	109/93	90/87	74/85	30/31	33/34	0.72
Age Mean ± SD (years)	48.5 ± 19	47.1 ± 20	51.9 ± 17	47.5 ± 17	47.4 ± 19	0.27
Smoking %	5	7	12	8	9	0.26
DM %	10	14	13	16	16	0.35
HTN %	24	23	19	21	27	0.77
IHD %	4	5	6	2	6	0.66
Tinnitus %	75	66	69	66	61	0.18
Vertigo %	35	36	30	30	19	0.12

The *p*-value represents the difference between half, one and 2 weeks vs. three and 4 weeks and over. M/F, male/female; DM, Diabetes Mellitus; HTN, Hypertension; IHD, Ischemic Heart Disease. *p*-value obtained from Kruskal-Wallis or ANOVA test as appropriate.

The average period of HL from symptom onset to initiation of treatment was 10.8 days. Most patients presented within the first two weeks (538, 80%), while 61 patients (9%) presented more than 4 weeks after symptom onset (Table 1). Following 1 week of oral treatment, 283 patients showed no or only very mild improvement and continued for another week of intratympanic treatment.

**Figure 1:**
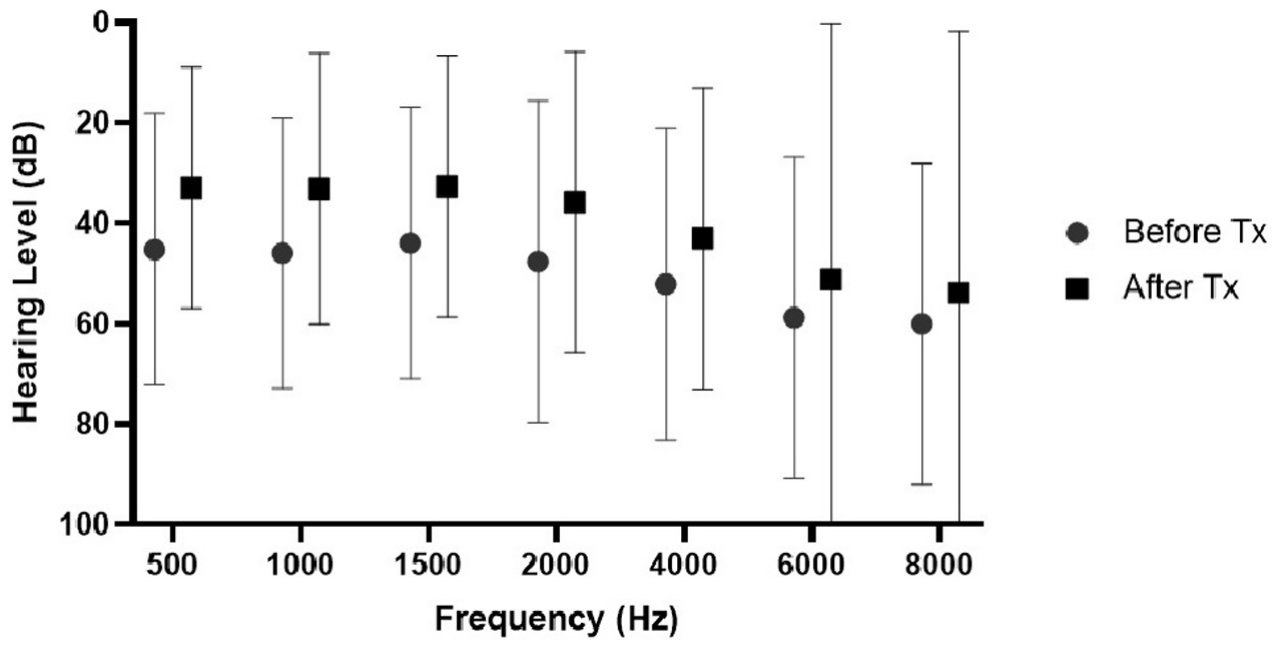
Hearing improvement following treatment according to the various frequencies of all 666 patients. Values represent mean and standard deviation.

The results of the first hearing test according to the different treatment groups are presented in Table 2. A statistically significant difference was found in both absolute and relative SRT, PTA, and speech discrimination between the different groups. Figure 1 shows hearing improvement in pure tone audiometry before and at the end of the treatment. No significant difference in improvement was found between the examined frequencies.

**Table 2:** Primary audiometry results according to treatment initiation time in weeks from symptom onset.

Weeks	0.5	1	2	3	4+	*p*
Number	202	177	159	61	67	
First test SRT dB	59.2 ± 35	62.2 ± 34	48.8 ± 32	38.9 ± 26	44.1 ± 28	**<0.001**
First test PTA dB	52.3 ± 30	52.4 ± 29	39 ± 25	37.5 ± 20	39.9 ± 23	**<0.001**
First test discrimination %	51.4 ± 43	50.7 ± 44	67.4 ± 39	76.6 ± 33	75 ± 33	**<0.001**
Healthy ear - affected ear. First test SRT dB	43.4 ± 33	44.5 ± 34	31.4 ± 27	25.3 ± 25	28.4 ± 25	**<0.001**
Healthy ear - affected ear. First test PTA dB	37.4 ± 28	37.7 ± 29	26.2 ± 18	26.9 ± 18	27.4 ± 20	**<0.001**
Healthy ear - affected ear discrimination %	45.2 ± 43	42.3 ± 43	26.5 ± 36	22.4 ± 33	21.6 ± 31	**<0.001**

Bold values: *p* < 0.05. Mean ± SD. The *p*-value (Kruskal-Wallis) represents the difference between half, one and 2 weeks vs. 3 and 4 weeks and over. SRT, Speech Recognition Threshold; dB, Decibels; PTA, Pure Tune Average.

Hearing improvement at the end of the treatment according to the different treatment groups is presented in Table 3. After Bonferroni correction, statistically significant differences were found between the groups of half a week, one week, and 2 weeks, and the groups of 3 weeks and 4 weeks and over (each group independently) in SRT, PTA, and discrimination. No difference was found between half a week, one week, and 2 weeks. No difference was also found between the three-week group and the 4 weeks and above group (Table 3).

**Table 3:** Hearing improvement indicators at the end of the treatment according to treatment initiation time in weeks from symptom onset.

Weeks	0.5	1	2	3	4+	*p*
Number	202	177	159	61	67	
SRT improvement dB	24.6 ± 23	23.4 ± 24	16.1 ± 17	5.8 ± 13	4.8 ± 7	**<0.001**
SRT relative improvement	0.53 ± 0.4	0.45 ± 0.4	0.41 ± 0.3	0.13 ± 0.5	0.2 ± 0.3	**<0.001**
PTA improvement dB	22.9 ± 24	19.8 ± 22	14.5 ± 13	5.5 ± 12	4.5 ± 8	**<0.001**
PTA relative improvement	0.52 ± 0.5	0.43 ± 0.5	0.4 ± 0.4	0.18 ± 0.4	0.24 ± 0.4	**<0.001**
Discrimination improvement %	30 ± 34	26 ± 33	18 ± 27	13 ± 18	7 ± 10	**<0.001**
Discrimination relative improvement	0.57 ± 0.4	0.52 ± 0.5	0.53 ± 0.4	0.51 ± 0.3	0.41 ± 0.5	0.3

Bold values: *p* < 0.05. Mean ± SD. The *p*-value (Kruskal-Wallis) represents the difference between half, one and 2 weeks vs. three and 4 weeks and over. SRT, Speech Recognition Threshold; dB, Decibels.

**Figure 2:**
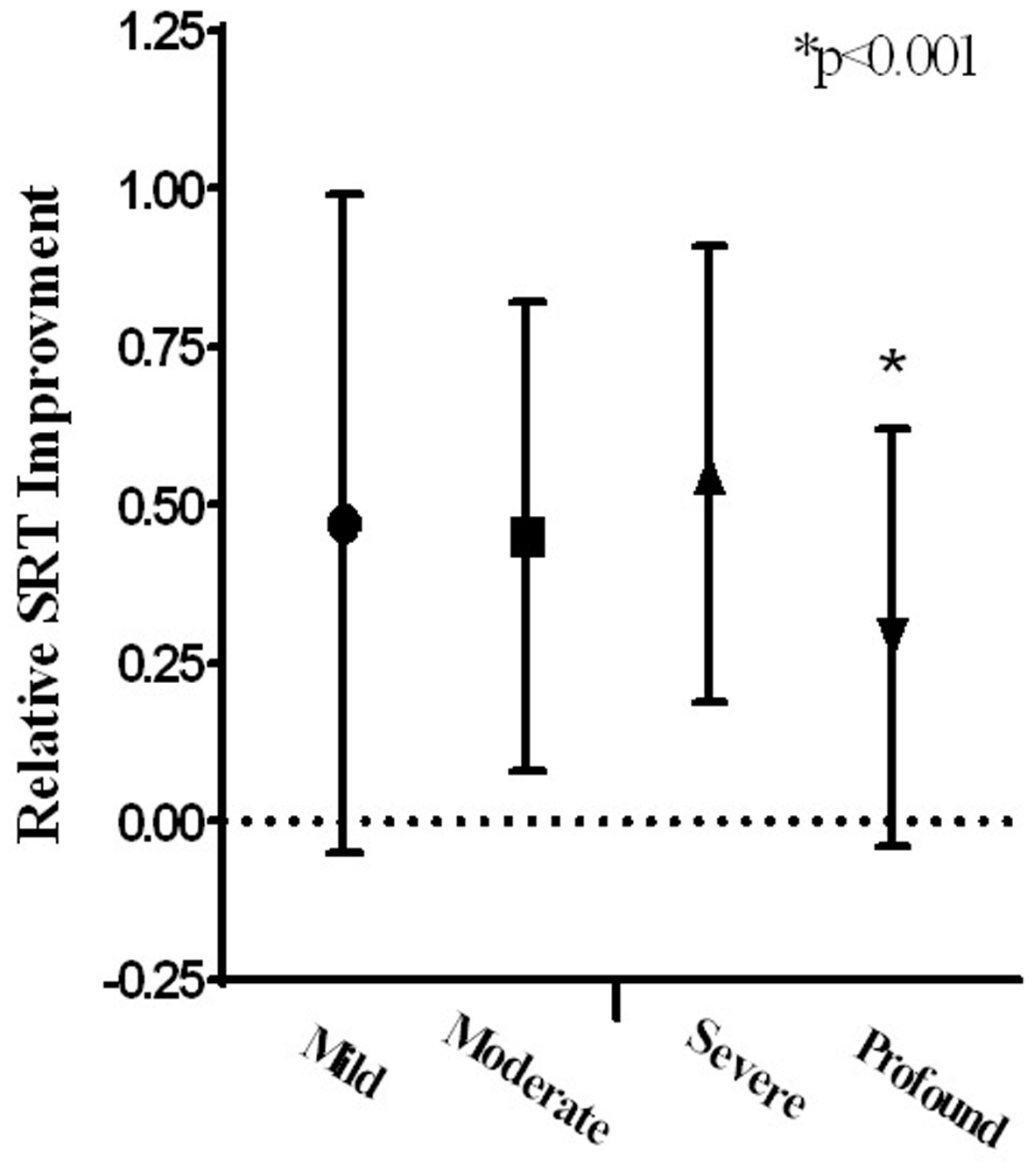
Relative SRT improvement according to hearing loss severity.

Table 4 presents the results of hearing improvement (in SRT) with respect to the severity of HL in the different treatment groups. The severity of HL was divided as follows: Mild HL (20–40 dB), Moderate and Moderate to Severe HL (40–70 dB), Severe HL (75–90 dB), and Profound HL (95–110 dB). According to the hearing loss severity, subgrouping analyses were made between time groups regarding gender, age, vascular risk factors (IHD, DM, HTN, smoking), and accompanying symptoms (tinnitus and vertigo). There was no statistical significance except for HTN in severe hearing loss, which also did not survive the Bonferroni correction (data not presented). Even after stratifying the results according to the severity of HL, the clear trend of significance is maintained between initiating treatment within 2 weeks of symptom onset and initiating treatment more than 2 weeks after symptom onset. A significant difference was found regarding relative SRT improvement between severity groups for all patients (Mild: 0.47 ± 0.5, Moderate: 0.45 ± 0.4, Severe: 0.54 ± 0.4, Profound: 0.29 ± 0.3, *p* < 0.001). After Bonferroni correction, statistically significant differences were found between Profound and each of the other three groups: Mild (*p* < 0.001), Moderate (*p* = 0.02), and Severe (*p* = 0.001). No difference was found between Mild, Moderate, and Severe (Figure 2).

**Table 4:** Hearing improvement indicators at the end of the treatment according to treatment initiation time in weeks from symptom onset and hearing loss severity.

Hearing loss	Weeks	0.5	1	2	3	4+	*p*
Mild	Number	87	73	89	39	44	
	SRT improvement dB	10 ± 10	10 ± 10	8 ± 7	3 ± 8	5 ± 6	**<0.001**
	SRT relative improvement	0.63 ± 0.5	0.54 ± 0.2	0.49 ± 0.4	0.22 ± 0.6	0.29 ± 0.4	**<0.001**
Moderate	Number	46	35	37	15	12	
	SRT improvement dB	26 ± 18	19 ± 18	15 ± 13	9 ± 14	5 ± 8	**<0.001**
	SRT relative improvement	0.62 ± 0.3	0.43 ± 0.3	0.43 ± 0.3	0.25 ± 0.4	0.13 ± 0.2	**<0.001**
Severe	Number	24	21	9	2	6	
	SRT improvement dB	44 ± 25	35 ± 27	36 ± 15	20 ± 14	5 ± 6	**<0.001**
	SRT relative improvement	0.68 ± 0.3	0.54 ± 0.4	0.62 ± 0.2	0.31 ± 0.2	0.16 ± 0.2	**<0.001**
Profound	Number	42	43	20	4	4	
	SRT improvement dB	35 ± 26	34 ± 30	27 ± 30	5 ± 15	1 ± 0.25	**<0.001**
	SRT relative improvement	0.43 ± 0.3	0.4 ± 35	0.31 ± 0.3	0.5 ± 0.1	0.1 ± 0.3	**<0.001**

Bold values: *p* < 0.05. Mean ± SD. The *p*-value (Kruskal-Wallis) represents the difference between half, one, and 2 weeks vs. three and 4 weeks and over. SRT, Speech Recognition Threshold; dB, Decibels.

A statistically significant correlation was found between treatment initiation in days (from symptom onset) and improvement in absolute and relative SRT and PTA indices (*R* = 0.23, *p* < 0.001, *R* = 0.11, *p* < 0.019, respectively).

No correlation was found between improvement in absolute and relative SRT indices and gender (*p* = 0.2 and *p* = 0.18, respectively) or age (*p* = 0.32 and *p* = 0.22, respectively). Furthermore, no correlation was found between vascular risk factors and improvement in absolute and relative SRT indices: DM (*p* = 0.22 and *p* = 0.16, respectively), IHD (*p* = 0.12 and *p* = 0.1, respectively), HTN (*p* = 0.81 and *p* = 0.62, respectively) and smoking (*p* = 0.61 and *p* = 0.45, respectively).

No correlation was found between improvement in absolute and relative SRT indices and the accompanying symptoms, tinnitus (*p* = 0.86 and *p* = 0.89, respectively) and vertigo (*p* = 0.85 and *p* = 0.43, respectively). Similarly, PTA and discrimination (absolute and relative) were not found to be in correlation with demographic parameters, vascular risk factors, or accompanying symptoms (data not presented).

In 65 patients, HL was in the high frequencies (above 30,000 Hz), with no loss of hearing in the lower and middle frequencies (Healthy SRT - AFFbef SRT = 0). The same trend of improvement described above was demonstrated; however, it was not significant due to the low numbers.

## 4. Discussion

In this study, we found an effect of the time from onset of SSNHL symptoms to treatment initiation on the amplitude of hearing improvement. A correlation of 20% was found between the time to treatment initiation and the degree of improvement in dB. Moreover, we found that the window of opportunity lasts up to 14 days, beyond which the effectiveness of the treatment decreases significantly. In addition, there is a clear, but not significant, trend of decreasing treatment efficacy from half a week to 2 weeks. Even after stratifying the results according to the severity of HL into four groups (mild, moderate, severe, and profound), the drop in treatment effectiveness after 2 weeks and the non-significant trend within the first two weeks are clearly maintained in each HL severity group. We found that tinnitus, vertigo, age, and patient medical background (IHD, DM, HTN) were not prognostic factors for hearing improvement.

This study is the first to examine the recommendations of the AAO, Head and Neck Surgery, published in 2012 and updated in 2019 (7, 11). For the first time, the recommendation to proceed to salvage treatment of intratympanic injection (ITI) with Dexamethasone after the failure of oral corticosteroid treatment (OCT) was examined. All patients included in this study were treated with Prednisone 60 mg daily for a week (except for patients whose blood sugar level and blood pressure were not balanced; in those cases, ITI was the initial treatment). In patients where a significant hearing improvement (baseline or close to baseline) was noticed at the end of the week with OCT treatment, another week of OCT tapper-down treatment was given. If no improvement was seen or slight improvement only, a laser myringotomy followed by ITI once daily with Dexamethasone was initiated for another week. The results of the hearing tests at the end of the treatment, whether they ended with OCT or ITI, were compared to the results of the first hearing test.

There is an inherent and significant difficulty involved in comparing the severity of HL and, consequently, the degree of improvement among different patients. In most cases, the patient’s specific hearing threshold before the HL is unknown. Hence, the severity of HL and the consequent improvement cannot be determined. To overcome this problem, and assuming that in most patients, the hearing was symmetrical before the onset of unilateral HL (unless otherwise known), we used the results of the hearing test of the healthy ear as a reference for the condition of the diseased ear before the HL (10). We found a reference to this approach in the paper by Cvorovic et al., who used it the same way. It should be noted that the improvement in hearing is presented according to this method (relative) as well as in absolute terms (comparing before and after the affected ear).

Over the years, several retrospective studies have attempted to find prognostic factors for improvement concerning SSNHL. Among all the factors tested, the effect of the time from onset of HL to initiation of treatment was also tested. Fetterman et al. examined 184 patients treated with corticosteroids and found no correlation between time to initiation of treatment and improvement in hearing (9). In contrast, Byl et al., in a study published in 1977 that is considered a cornerstone in SSNHL research, found that for 26 patients treated with CTS, the treatment was effective if given up to 10 days after the onset of HL (3). Similarly, Change et al. found a worse prognostic factor among 146 patients starting treatment after the sixth day than among those starting treatment earlier (12). Xenellisand et al. also found a significant correlation between the time to initiation of treatment and improvement in hearing among 114 patients. Still, they did not specify the time point after which there was a substantial decrease in improvement (13). In a study on 541 patients, Cvorovic et al. found a significant difference in the degree of improvement in hearing if the treatment began more than 7 days after the onset of HL. The treatment given in this study was Prednisone 100 mg once a day for 7 days with no ITI treatment (10).

Despite accepted assumptions regarding the importance of the time to treatment initiation, more well-founded information is required to support this assumption. Moreover, there is a dispute as to what, if any, time period is relevant. In addition, the extent of the effect of the time to treatment initiation has never been examined with respect to the new recommendations, which include treatment with Prednisone 60 mg per day for 7 days and the addition of ITI treatment in case of a lack of improvement (11). In this study, for the first time, we found the precise time point (14 days) at which there is a significant drop in hearing gain following CTS treatment.

When examining the severity of hearing loss among the different time groups, it appears that patients who started treatment later presented with milder hearing loss, both in SRT and in discrimination, compared to patients who began treatment earlier (Table 2). We can consider several explanations for this phenomenon. The degree of urgency to receive treatment may be lower for patients who suffer from milder HL since the HL is less noticeable. On the other hand, this trend may reflect a natural healing process over time, regardless of treatment. It can be assumed that patients who arrived after 2 weeks suffered initially from a more significant decrease in hearing that gradually improved over time. The average SRT results before treatment among the group that presented during the third week (group four: 61 patients) are similar to the results at the end of treatment for the groups that presented during the first week (groups one and two: 379 patients), 38.9 ± 26 and 36.7 ± 23, respectively. In other words, at the same time point since the onset of HL, the results were similar whether the patients received treatment or not. Therefore, we can conclude that the improvement of the early group is not a result of the CTS treatment but rather reflects the natural process of hearing improvement that took place in the group that presented later. A Cochrane review from 2013 concluded that the value of CTS in treating SSNHL remains unclear. The evidence obtained from randomized controlled trials presents contradictory outcomes, partly because the studies are based on an inadequate number of patients (14). These findings raise the question of whether CTS treatment helps at all.

No significant effects were found in the current study for any of the tested cardiovascular risk factors (CVRF): age, DM, IHD, HTN, and smoking. Previous studies have indicated that traditional cardiovascular risk factors such as HTN, DM, smoking, and IHD may contribute to SSNHL and have an impact on HL improvement (4, 10, 13, 15–18). However, these studies’ weaknesses remain in their retrospective analysis, relatively small population sizes, and univariate analysis. Along the same line as our findings, Ullrich et al. and Ballesteros et al. found an identical frequency of CVRFs between controls and SSNHL patients (19, 20). Moreover, a meta-analysis recently published found that only hypercholesterolemia may be an independent risk factor for SSNHL but not other CVRF. They conclude that to clarify the relation between CVRFs and SSNHL, long-term, multi-center, and prospective studies are crucial but challenging (21). As with CVRF, the relationship between vertigo and tinnitus to severity and improvement in SSNHL is still controversial. An association between vertigo and poor auditory recovery prognosis has been observed (4, 12, 22, 23). Several theories have been described to explain this finding, including rupture of the labyrinthine membranes (24), the degree of biochemical alterations in the labyrinthine ionic composition (25), and the association with vestibular neuritis (26). However, as with our findings, in multivariate analysis, vertigo was not significantly associated with a worse hearing recovery prognosis (27). We believe that the strength of our study lies in the high number of patients included. Therefore, it further contributes to clarifying these doubtful questions.

This study has several limitations. First, it is a retrospective study and does not include a comparison with a control group. Of course, it is impossible to conduct such an analysis for ethical reasons since the accepted treatment worldwide is CTS, despite the absolute lack of evidence. In addition, among all patients presenting with SSNHL, there are many subgroups: HL at different frequencies, severity of HL, etc. Therefore, there is an inherent difficulty in including them all in one group. In this study, stratification was carried out as much as possible for these parameters. However, it is never possible to go down to the lowest resolution because this will create many groups with a small number of patients, affecting the statistical power of the analysis. Since this study’s final cohort includes a large number of patients (666)—a larger sample than in all the studies carried out to date—we can assume that our results have critical statistical and clinical significance. Another limitation is the short-term follow-up since the last hearing test was conducted at the end of the treatment. However, this study aimed to compare treatment time groups at this time point, and we found significant differences. Moreover, most hearing improvements happened in the first few weeks (parallel to the end of treatment). No significant improvement or deterioration has been found in the long term, neither after several months nor after several years (28, 29).

## 5. Conclusion

The time from onset of HL to initiation of treatment is a prognostic factor with a correlation of *ca.* 20% with the degree of improvement in hearing. No significant trend was found within the first 14 days from the onset of HL. After 14 days, the effectiveness of the treatment drops dramatically. Age, accompanying symptoms (tinnitus, vertigo), smoking, and underlying diseases (IHD, DM, HTN) are not prognostic factors for hearing improvement and the success of CTS treatment.

## Data availability statement

The raw data supporting the conclusions of this article will be made available by the authors, without undue reservation.

## Author contributions

IC helped design the research, performed the research, analyzed the data, and took part in writing the manuscript. SE and OM performed the research and helped in data analysis. RS took a significant part in revising the paper. J-YS and RP helped in designing the study and writing of the manuscript. CS designed the study, analyzed the data, and drafted the paper. All authors contributed to the article and approved the submitted version.

## Conflict of interest

The authors declare that the research was conducted in the absence of any commercial or financial relationships that could be construed as a potential conflict of interest.

## Publisher’s note

All claims expressed in this article are solely those of the authors and do not necessarily represent those of their affiliated organizations, or those of the publisher, the editors and the reviewers. Any product that may be evaluated in this article, or claim that may be made by its manufacturer, is not guaranteed or endorsed by the publisher.
